# Native Pyroglutamation of Huwentoxin-IV: A Post-Translational Modification that Increases the Trapping Ability to the Sodium Channel

**DOI:** 10.1371/journal.pone.0065984

**Published:** 2013-06-24

**Authors:** Mingqiang Rong, Zhigui Duan, Juliang Chen, Jianglin Li, Yuchen Xiao, Songping Liang

**Affiliations:** 1 The Key Laboratory of Protein Chemistry and Developmental Biology of Ministry of Education, College of Life Sciences, Hunan Normal University, Changsha, China; 2 Key Laboratory of Animal Models and Human Disease Mechanisms of Chinese Academy of Sciences and Yunnan Province, Kunming Institute of Zoology, Kunming, Yunnan, China; Virginia Commonwealth University, United States of America

## Abstract

Huwentoxin-IV (HWTX-IV), a tetrodotoxin-sensitive (TTX-s) sodium channel antagonist, is found in the venom of the Chinese spider *Ornithoctonus huwena*. A naturally modified HWTX-IV (mHWTX-IV), having a molecular mass 18 Da lower than HWTX-IV, has also been isolated from the venom of the same spider. By a combination of enzymatic fragmentation and MS/MS *de novo* sequencing, mHWTX-IV has been shown to have the same amino acid sequence as that of HWTX-IV, except that the N-terminal glutamic acid replaced by pyroglutamic acid. mHWTX-IV inhibited tetrodotoxin-sensitive voltage-gated sodium channels of dorsal root ganglion neurons with an IC_50_ nearly equal to native HWTX-IV. mHWTX-IV showed the same activation and inactivation kinetics seen for native HWTX-IV. In contrast with HWTX-IV, which dissociates at moderate voltage depolarization voltages (+50 mV, 180000 ms), mHWTX-IV inhibition of TTX-sensitive sodium channels is not reversed by strong depolarization voltages (+200 mV, 500 ms). Recovery of Nav1.7current was voltage-dependent and was induced by extreme depolarization in the presence of HWTX-IV, but no obvious current was elicited after application of mHWTX-IV. Our data indicate that the N-terminal modification of HWTX-IV gives the peptide toxin a greater ability to trap the voltage sensor in the sodium channel. Loss of a negative charge, caused by cyclization at the N-terminus, is a possible reason why the modified toxin binds much stronger. To our knowledge, this is the first report of a pyroglutamic acid residue in a spider toxin; this modification seems to increase the trapping ability of the voltage sensor in the sodium channel.

## Introduction

Spider venom is a complex mixture of components which exhibit a diverse array of actions both on prey and on human victims [1]. Previous research has identified nearly 150 of these components in the Chinese bird spider, *Ornithoctonus huwena* [2], which is one of the most venomous spiders in China [3]. Most of the toxins have six cystine residues adopting inhibitor cystine-knot (ICK) motif with a 1–4, 2–5, 3–6 disulfide bonding pattern; the biological activities of some peptide toxins were thoroughly investigated. As a group, the toxins possess quite different biological activities, including inhibition of voltage-gated calcium and sodium channels, insecticidal activity, lectin-like agglutination, and inhibition of trypsin [4]–[8]. Huwentoxin-IV (HWTX-IV), similarly to JZTX-34 [9], inhibited neuronal TTX-sensitive voltage-gated sodium channels with an IC_50_ value of 30 nM in adult rat dorsal root ganglion neurons (DRG neurons), but have no significant effect on TTX-resistant voltage-gated sodium channels. Recently studies demonstrated that ProTx-II and HWTX-IV binding determinants on domain-II may overlap, with domain II playing a much more crucial role for HWTX-IV [10]. The date also proved that the inhibition of sodium currents could be reversible by strong depolarization due to the dissociation of HWTX-IV [11], [12].

A variety of posttranslational modifications of peptide toxins have been identified and characterized. These include amidation of the C-terminus, epimerization to a D-amino acid, *O*-glycosylation of Ser and Thr, disulfide formation, γ-carboxylation of Glu, bromination of Trp, cyclization of Gln, sulfation of Tyr, and hydroxylation of Pro. Some posttranslational modifications are easily predicted as being due to proteolytic processing, e.g., C-terminal amidation and disulfide bond formation [13], [14]. Others are unusual or rare modifications in peptides are detected by standard proteomic methods such as MS or Edman sequencing [15]. The molecular diversity of these toxins is enhanced by the posttranslational modification. It has been assumed that some posttranslational modifications improve the function [16]. For example, the peptide with D-Phe44 but not the L-Phe is highly potent in eliciting the repetitive activity; Contulakin-G, which contains glycosylation, shows ten folds activity more than no-glycosylation toxin [17], [18].

Up to now, most of posttranslational modifications are found in conotoxin, there are only rare report of modified spider toxins except C-terminal amidation and disulfide bond formation. In present work, we described the purification and identification of a new modification in a toxin from the Chinese bird spider, *Ornithoctonus huwena*. In order to know the difference between HWTX-IV and mHWTX-IV, we used mass spectrometry to indentify the posttranslational modification and whole cell recording to investigate the function on sodium channel.

## Experimental Procedures

### Toxin Purification and Molecular Mass Determination

The venom from the female Chinese bird spider (*S. Huwena*) was collected as described in our previous work [6]. Toxins were purified by means of ion-exchange and reverse-phase high performance liquid chromatography. Lyophilized crude venom was loaded onto a Waters Protein- Pak CM 8HR ion-exchange column (5×50 mm) initially equilibrated with 0.02 M sodium phosphate buffer, pH 6.25 (buffer A). The column was eluted with a linear gradient of 0–50% of buffer B (1 M sodium chloride, 0.02 M sodium phosphate, pH 6.25) over 80 min at a flow rate of 3 ml min^−1^. The fraction of interest was then applied to a Vydac C18 analytical reverse-phase HPLC column (218TP54, 4.6×250 mm) and eluted at a flow rate of 1 ml/min using a gradient of 0–10% buffer B (0.1% v/v trifluoroacetic acid in acetonitrile) over 8 min. After an equilibrium period of 2 min, a gradient of 10–50% buffer B over 40 min was used. (Buffer A was 0.1% v/v trifluoroacetic acid in water.) Further purification was applied in the same equipment and column at a flow rate of 1 ml/min using a gradient of 0–20% buffer B (0.1% v/v trifluoroacetic acid in acetonitrile) over 8 min. A gradient of 28–40% buffer B over 30 min was followed by an equilibrium period of 2 min. Once purified to >99% homogeneity (assessed by reverse-phase HPLC and mass spectrometry), peptide was lyophilized and stored at −20°C until further use.

### Molecular Mass Determination and Peptide sequencing

The molecular mass was determined by matrix-assisted laser desorption or ionization time-of-flight mass spectrometry (MALDI- TOF MS) on a Bruker ProFlex-III mass spectrometer. Amino acid sequence of purified neurotoxin was determined by Edman degradation using a Applied Biosystems/PerkinElmer Life Sciences Procise 491-A protein sequencer.

### Digestion

For direct digestion, 200 μg of toxins were dissolved in 20 μL of 0.5% SDS buffer and boiled for 3 min. The proteins in the sample were reduced with 10 mM DTT at 56°C for 1 h and half of toxin alkylated by 55 mM IAA in the dark at room temperature for 45 min. The alkylated toxin and unalkylated toxin were diluted to 200 μL with a solution containing 30 mM NH_4_HCO_3_. Finally, tryptic digestion was carried out by 4 μg of trypsin and incubation at 37°C for 20 h. The enzymatic digestion was stopped by acidification using diluted acetic acid.

### CapLC-MS/MS Analysis

Tryptic peptides obtained from the fractionation of direct digestion of the toxins were separately injected into a capillary LC system (Waters) and first desalted and preconcentrated on a C18 PepMapTM precolumn (0.3 mm ×5 mm; LC Packings). The peptides were then eluted onto a C18 column (75 μm ×15 cm; LC Packings, Sunnyvale, CA) coupled to a quadrupole time-of-ﬂight (Q-TOF) microhybrid mass spectrometer (Q-TOF microTM, Waters/Micromass, Manchester, UK) equipped with a micro mass nano-ESI source. The tryptic peptide was eluted at a linear gradient from 5% to 50% B (0.1% formic acid/4.9% H_2_O/90% ACN, v/v/v) over 65 min and then followed by a 10 min gradient to 85% B. Finally, the gradient increased to 95% B over 10 min. The ﬂow rate was 2 μL/min. Eluted peptides were detected in positive ion MS mode and data-dependent MS/MS mode. The data-dependent mode was used for survey scans (m/z 100–1400) to choose up to three most intense precursor ions (with charge states ≥2). For collision-induced dissociation mass spectrometric (MS/MS) analysis, collision energy was chosen automatically as a function of m/z and charge. Collision gas was argon, the temperature of heated sample source was 85°C and electrospray voltage was 3,000 V.

### Transient Transfection

Nav1.7 channel plasmid and a plasmid for green fluorescent protein were transiently transfected into human embryonic kidney 293 (HEK293) cells by using the lipofectamine 2000 (Invitrogen, USA) and following manufacture's instruction. HEK293 cells were grown under standard tissue culture conditions (5% CO_2_; 37°C) in Dulbecco's modified Eagle's medium supplemented with 10% fetal bovine serum [19]. β1 subunit were cotransfect with Nav1.7 channel in order to increase the current density.

### Cell Isolation Procedures

30-day-old adult Sprague-Dawley rats of either sex were decapitated. Then the dorsal root ganglia were isolated quickly from the spinal cord [20].The dissociated cells were suspended in essential Dulbecco's modified Eagle's medium containing trypsin (0.5 g/L, type III), collagenase (1.0 g/L, type IA), and DNase (0.1 g/L, type III) to incubate at 34°C for 30 min. Trypsin inhibitor (1.5 g/L, type II-S) was used to terminate enzyme treatment. The DRG cells were transferred into 35-mmculture dishes (Corning, Sigma) containing 95% Dulbecco's modified Eagle's medium, 5% newborn calf serum, hypoxanthine aminopterin thymidine supplement, and penicillinstreptomycin and then incubated in the CO_2_ incubator (5% CO_2_, 95% air, 37°C) for 1–4 h before the patch-clamp experiment. TTX at 0.1 μM was added in bathing solution to separate TTX-resistant currents from mixture sodium currents on DRG neurons with smaller diameter. All of the experimental protocols using animals in this work were approved by the Animal Care and Use Committee at Hunan Normal University.

### Electrophysiological Studies

Cell currents were recorded on experimental DRG cells and HEK293 cells using whole-cell patch-clamp technique at room temperature (20–25°C). For sodium current recordings on DRG cells, the bath solution contained (in mM): 150 NaCl, 2 KCl, 5 D-glucose, 1 MgCl_2_, 1.5 CaCl_2_, and 10 HEPES at pH 7.4; the pipette internal solution contained (in mM): 105 CsF, 35 NaCl, 10 HEPES, and 10 EGTA at pH 7.4.

Cells with green fluorescent protein fluorescence in HEK293 cells were selected for whole-cell patch-clamp recording 36–72 h after transfection. Sodium currents were recorded using an internal solution containing (in mM): CsF 140, EGTA 1, NaCl 10, and HEPES 10, pH 7.3, and the external bathing solution contained (in mM): NaCl 140, KCl 3, MgCl_2_ 1, CaCl_2_ 1, and HEPES 10, pH 7.4.The patch pipettes with DC resistances of 2–3 M were fabricated from borosilicate glass tubing (VWR micropipettes,100 ml, VWR Company) using a two-stage vertical microelectrode puller (PC-10, Narishige, Japan) and fire-polished by a heater (Narishige, Japan). Whole-cell patch clamp technique by an Aoxn 700B patch clamp amplifier (AXON, American). The P/4 protocol was used to subtract linear capacitive and leakage currents. Experiments data were acquired and analyzed by using the program Clampfit10.0 (AXON, American) and Sigmaplot (Sigma).

### Data Analysis

Data were analyzed using the clampfit (Axon) and Sigmaplot9.0 (Sigma) software programs. All data points are shown as mean ± S.E. n stands for the number of the separate experimental cells. Dose-response curves were fitted using the following Hill logistic equation: y = 1-(1-fmax)/(1+([Tx]/IC_50_)^n^) where n is an empirical Hill coefficient and fmax is the fraction of current resistant to inhibition at high toxin (Tx) concentration.

## Results

### Peptide Purification and Molecular Mass Determination

**Figure 1:**
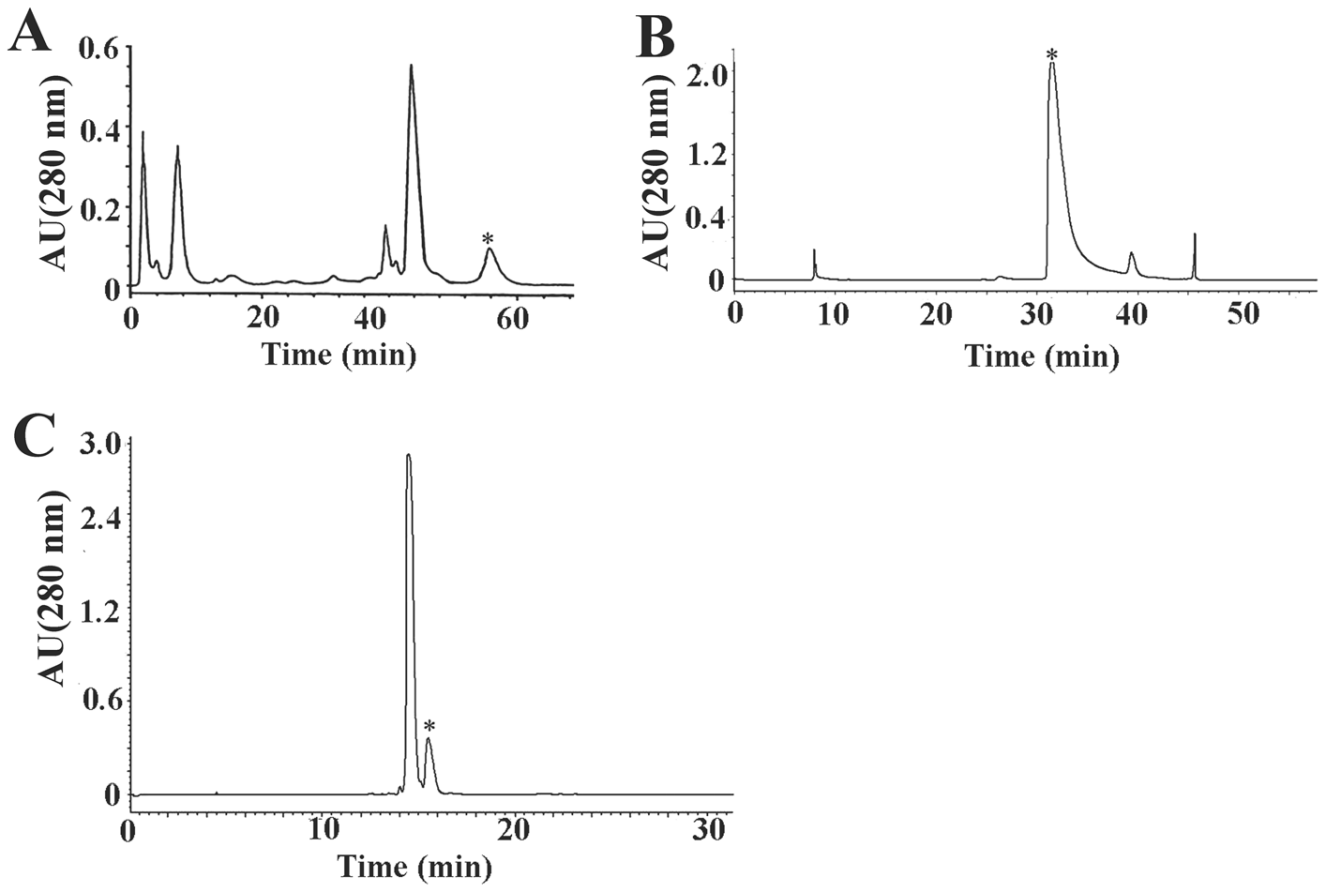
HPLC purification of mHWTX-IV. The peaks marked by * contain mHWTX-IV. (A) Elution profile of *Ornithoctonus huwena* Wang venom by ion-exchange HPLC. (B) Isolation of mHWTX-IV by RP-HPLC on a C18 column in a gradient of 10–50% acetonitrile over 50 min. (C) Further purification of mHWTX-IV by a repetitive RP-HPLC with a gradient of 28–40% acetonitrile over 30 min.

**Figure 2:**
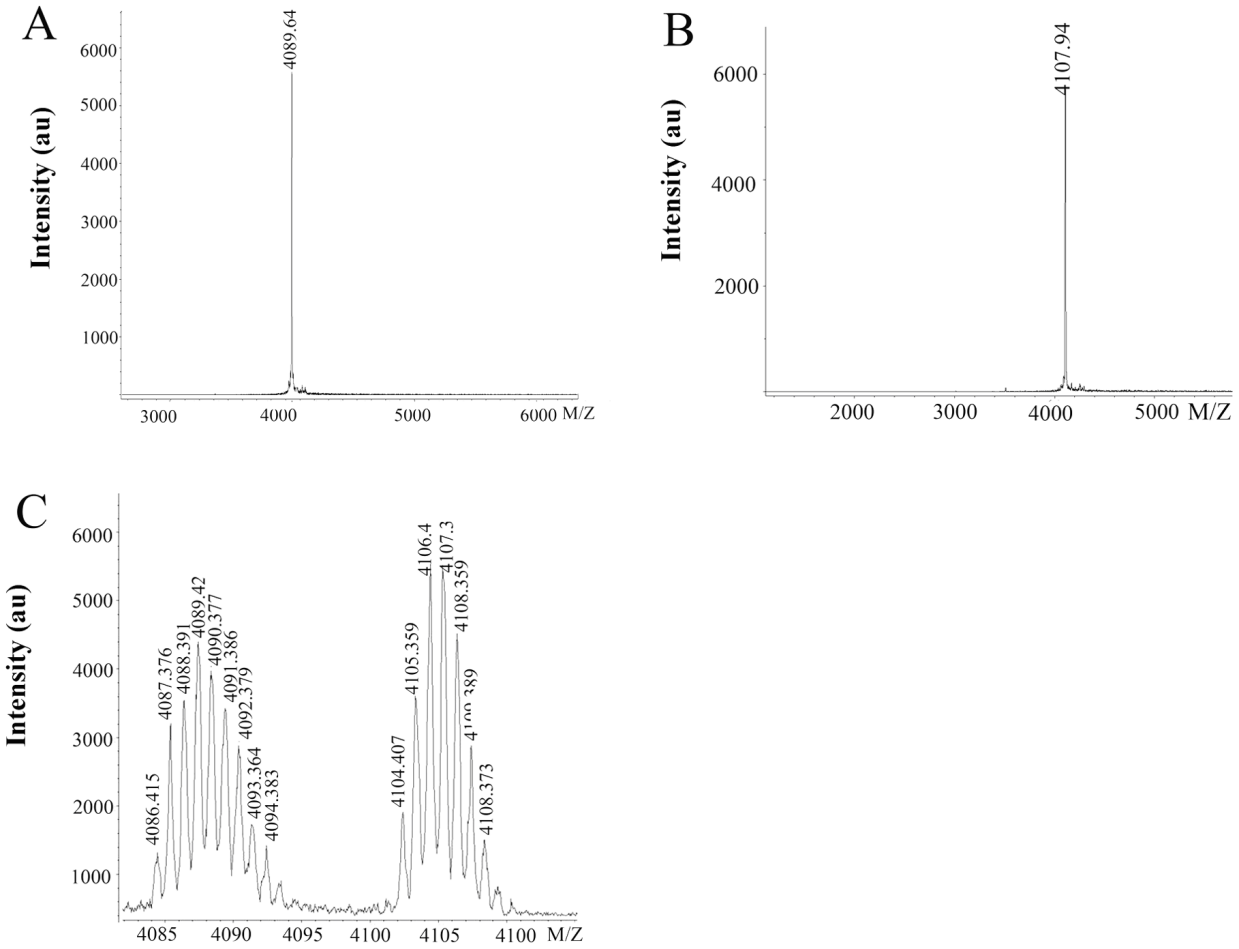
Mass spectrometry of mHWTX-IV and HWTX-IV. (A) Molecular mass of mHWTX-IV detected by mass spectrometry, 4089.64 Da. (B) Molecular mass of HWTX-IV, 4107.94 Da. (C) Monoisotopic mass spectrum of a mixture of mHWTX-IV and HWTX-IV.

The crude venom of Chinese bird spider, *Ornithoctonus huwena,* was separated into six peaks by ion-change HPLC as previous reported (Fig 1A). A peptide having a molecular mass of 4089.6 Da, 18 Da lower than that of native HWTX-IV (Fig 2A, B), was found to coelute with HWTX-IV using reverse-phase HPLC with a gradient of 10–50% buffer B over 40 min (Fig 1B). The two peptides could be separated on the same column using a gradient of 28–40% buffer B over 30 min, yielding the peptide, whose purity was determined to be over 99% by mass spectrometry.

In order to ascertain its molecular weight, the toxin was mixed with HWTX-IV and the two were analyzed by MALDI mass spectrometry. A cluster of signals was observed, but the first monoisotopic signal of the toxin was seen at m/z 4086.41, which corresponded to a monoisotopic molecular mass of HWTX-IV of 4104.40 (Fig 2C). This result demonstrated that the toxin had a mass 18 Da lower than that of HWTX-IV, presumably by loss of water, and was named “modified HWTX-IV” (mHWTX-IV), indicating that it is a posttranslational modified form of HWTX-IV. 0.2 mg HWTX-IV and mHWTX-IV were applied to detect the different amino acid sequence of the two toxins. The N-terminus sequence of HWTX-IV is composed by ECLEIF (Fig S1), while no signal of mHWTX-IV was detected (Fig S2). Since the N-terminal residue of HWTX-IV is glutamic aicd [21], [22], but no signal was detected on Edman degradation of mHWTX-IV, we proposed that the N-terminus of this peptide is pyroglutamic acid (pGlu), which accounts for the mass loss of 18 Da.

### Sequence and Posttranslational Modification Determination

**Figure 3:**
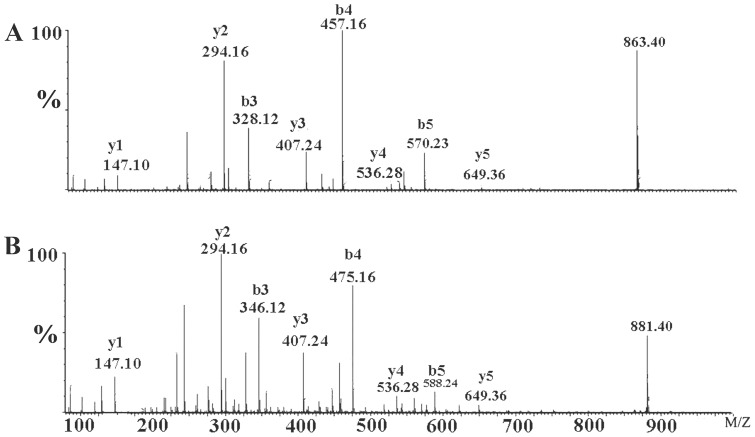
MS/MS spectrum for sequence determination of the first fragment of mHWTX-IV (A) and of HWTX-IV (B) after digestion with trypsin. The sequence was derived from the series of b-ions and y-ions.

**Figure 4:**
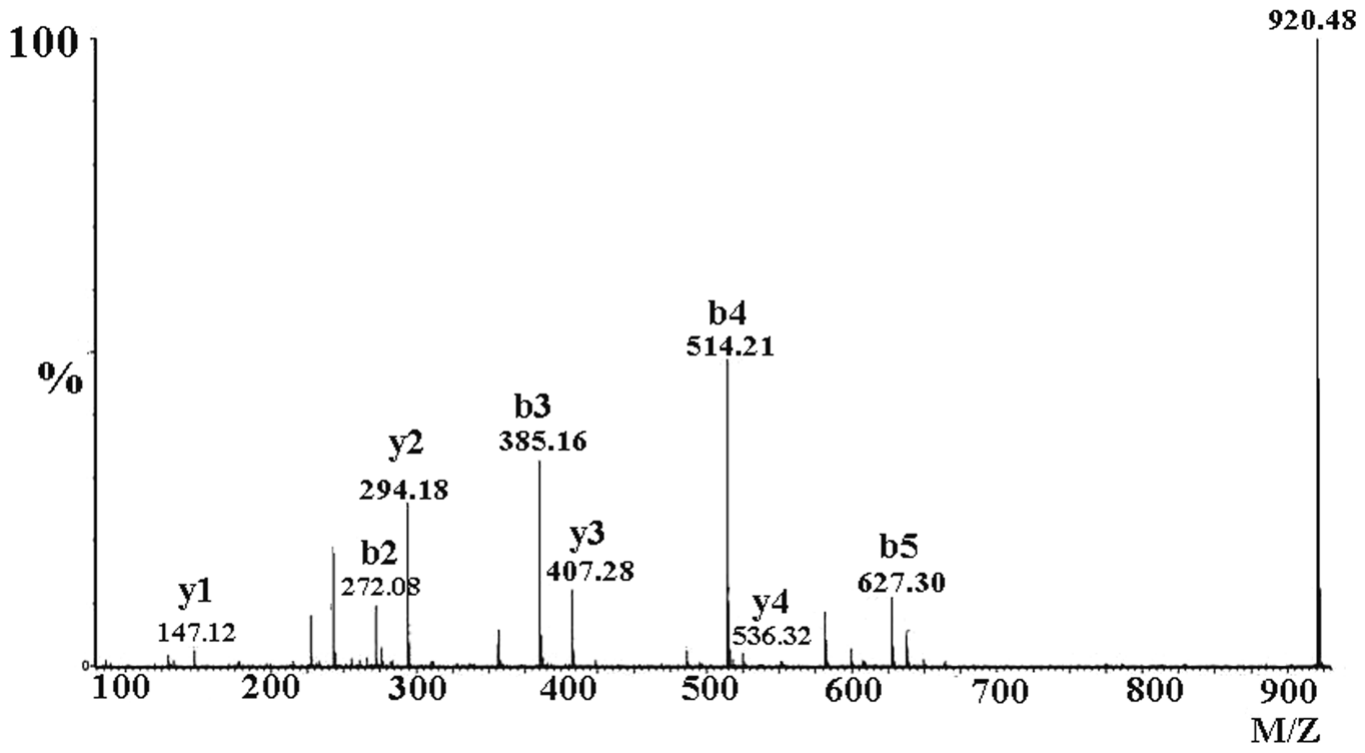
MS/MS spectrum for sequence determination of the N-terminal fragment of reduced and carboxamidomethylated mHWTX-IV. Sequence was derived from the series of b-ions and y-ions.

In order to further explore this possibility, mass spectrometry was used to deduce the sequence of the peptide and ascertain the position of posttranslational modification [23]. Since the toxin contains an ICK motif (three disulfide crosslinks), we cleaved the disulfiedes using dithiothreitol (DTT), subsequent trypsin digestion yielded six fragments. All fragments had the same molecular mass as the corresponding fragments from HWTX-IV, except that the first fragment of the modified peptide exhibited a mass 18 Da lower than that of HWTX-IV. This result also demonstrated that molecular weights of two peptides differ by 18 Da and that the modification was in the first fragment. To verify pyroglutamic acid at the N-terminus of mHWTX-IV, the first fragment was further analyzed using *de novo* sequencing. As shown in Fig. 3, most b-ions (b3,b4,b5) y-ions(y1,y2,y3,y4,y5) were detected and allowed for ready identification of the amino acids, LEIFK, in positions 3–7 (Fig. 3A,B). Furthermore, since the masses of the y-ions were the same for both peptides in these positions, the modification must have been in the first two amino acids, namely at glutamic acid or at cysteine. A specimen of mHWTX-IV was reduced and alkylated and further analyzed by mass spectrometry after digestion with trypsin. Reduction and alkylation of cysteine in the N-terminal fragment with iodoacetamide resulted in a mass increase of 57 Da as compared with the un-alkylated compound [24]. As shown in Fig. 4, the mass of b2 was 272.08 indicating that the sulfydryl had been modified by iodoacetamide. These results demonstrated that the sulfhydryl group of cysteine, at residue 2, was behaving normally (i.e., that it had been alkylated by iodoacetamide) and therefore that modification was likely at the N-terminal glutamic acid residue. The lack of reaction on Edman degradation, along with the molecular mass data, indicates that the peptide is pyroglutamic acid.

### Effects of mHWTX-IV on Sodium Channel

It has reported that HWTX-IV specifically inhibited the tetrodotoxin sensitive (TTX-S) voltage-gated sodium channel in DRG neurons [12], [21]. In order to know whether the modification of HWTX-IV results in alteration of its function, mHWTX-IV was also investigated on the voltage-gated sodium channel of DRG neuron.

**Figure 5:**
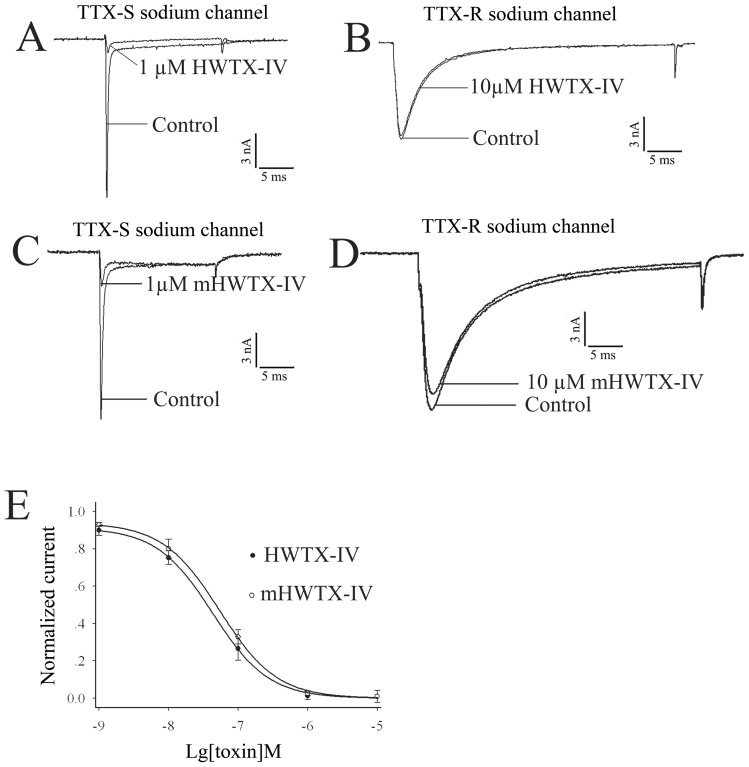
Effects of HWTX-IV and mHWTX-IV on sodium channel in rat DRG neurons. All current traces were evoked by a 50-ms step depolarization to −10 mV from a holding potential of −80 mV at every second. The currents of TTX-S were significantly reduced by 1-µM HWTX-IV (A) and 1-μM mHWTX-IV (C); 10-µM HWTX-IV (B) and 10-μM mHWTX-IV (D) had no effect on TTX-R sodium currents. (E) shows the concentration dependent inhibition of TTX-S sodium currents on DRG neurons by HWTX-IV (B) and mHWTX-IV. Control for each panel means no toxin treatment. Every data point (mean ± S.E) was obtained from five separate experimental cells.

Cells were held at −80 mV for over 4 min to allow adequate equilibration between the micropipette solution and the cell interior, and then the current traces were evoked using a 50 ms step depolarization to −10 mV every second. TTX at 0.1 μM was added in bathing solution to separate TTX-resistant currents from mixture sodium currents on DRG neurons with smaller diameter. As shown in Figure 5, modified and native HWTX-IV at concentration of 1 μM almost completely inhibited TTX-sensitive currents (Fig. 5A, C), but 10 μM mHWTX-IV and HWTX-IV showed no effect on TTX-R sodium channel (Fig. 5B, D). The rapid inhibition is dose-dependent with an IC_50_ value of 54.16±7.35 nM and 42.86±1.72 nM for mHWTX-IV and HWTX-IV, respectively (Fig. 5E). It implies that the posttranslational modification of HWTX-IV do not change the affinity to TTX-S sodium channel of DRG neuron.

**Figure 6:**
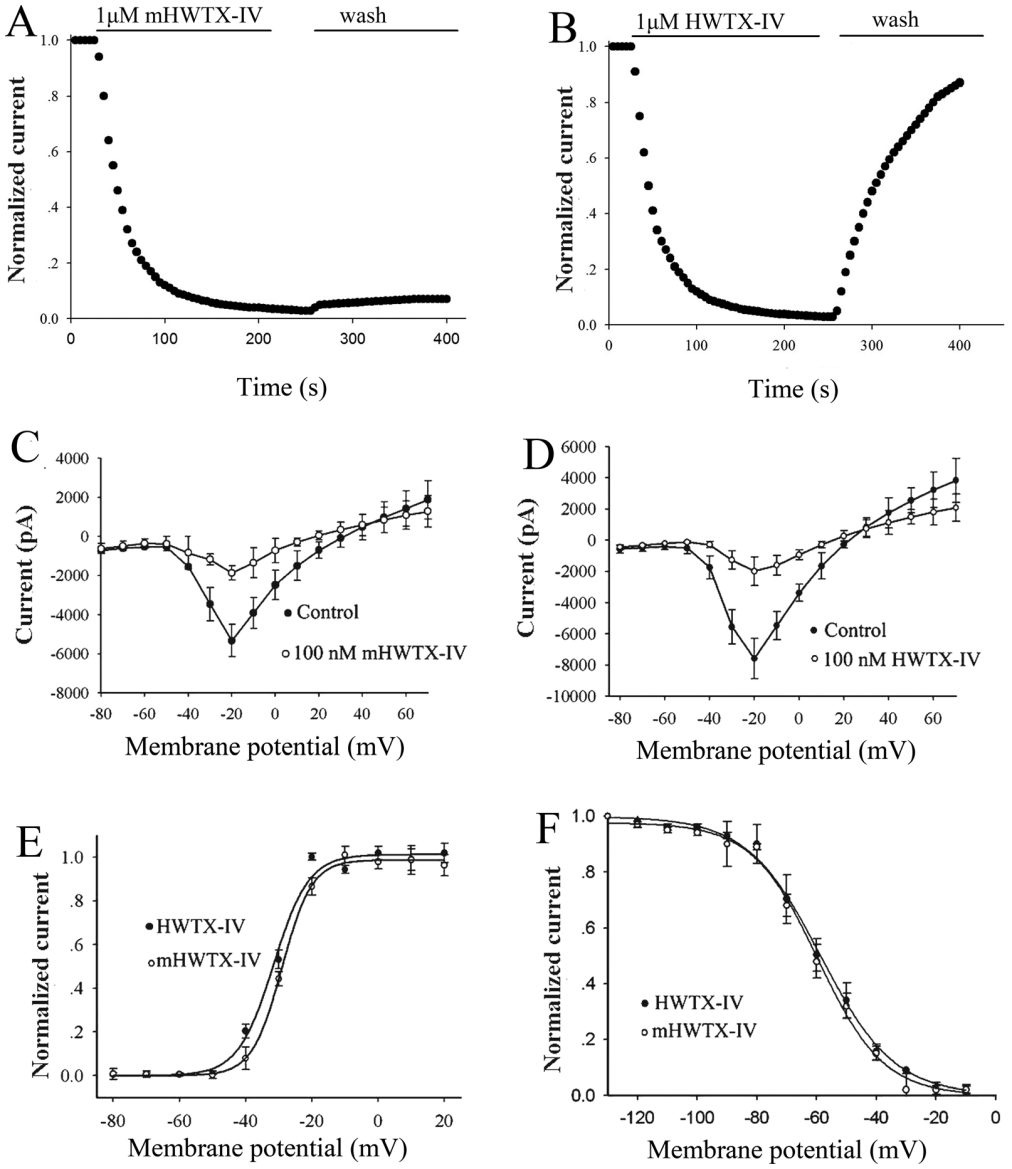
Effects of HWTX-IV and mHWTX-IV on the kinetics of TTX-S sodium channels in rat DRG neurons. Time course for block of TTX-S currents and reversal of block by mHWTX-IV (A) and HWTX-IV (B). Current-voltage (I–V) relationships of sodium currents before and after adding 100 nM mHWTX-IV (AC), HWTX-IV (BD). HWTX-IV and mHWTX-IV showed no obvious difference on the steady-state activation (CE) and inactivation (DF). Control for each panel means no toxin treatment. Every data point (mean ± S.E.) was obtained from five separate experimental cells. The data points for both activation and inactivation kinetics were well fitted with the Boltzmann equation.

The action of 1 μM toxins on TTX-s currents was fast, the time course for inhibition of mHWTX-IV and HWTX-IV was 27.8 s and 25.4 s, respectively (Fig. 6A,B). After washing with extracellular solution, HWTX-IV was slowly dissociated from sodium with the time course of 88.3 s (Fig. 6B). However, almost no dissociation was detected in the inhibition by 1 μM mHWTX-IV (Fig. 6A). Moreover, like native HWTX-IV, 100 nM mHWTX-IV did not change the threshold of activation and alter the reversal potential of TTX-S sodium channel (Fig. 6C, D). As observed on the conduct–voltage curve, two toxins did not change channel conductance at voltages varying from −80 to +20 mV (Fig. 6E). Therefore we investigated the effect of mHWTX-IV and HWTX-IV on steady-state inactivation of TTX-S sodium channel using a standard two-pulse protocol. No shift of the steady-state inactivation curve of TTX-S sodium channel was induced (Fig. 6F). These results suggest that there is no observable difference between mHWTX-IV and HWTX-IV when applied to the TTX-S sodium channel of DRG neuron.

**Figure 7:**
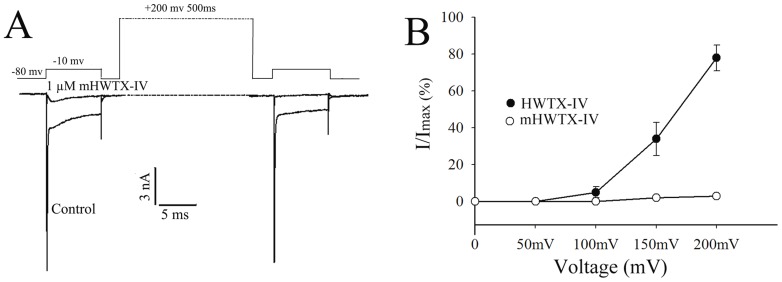
Sodium current recording after the application of mHWTX-IV detected by strong depolarization. (A) DRG neurons were held at −80 mV and then with a 50 ms test pulse of −10 mV. A +200 mV 500 ms strong depolarization applied after cell back held at −80 mV. Finally, a −10 mV pulse used to test the currents. After the +200 mV strong depolarization, no current was induced. (B) Recovery of current from Nav1.7 following strong depolarization in the presence of 1 µM HWTX-IV or 1 µM mHWTX-IV. HEK293 cells were depolarized to +200, +150, +100 and +50 mV. Control for each panel means no toxin treatment. Every data point (mean ± S.E.) was obtained from five separate experimental cells.

HWTX-IV like other site 4 toxins was dissociated by strong depolarization and currents can be observed at voltages above +70 mV [25], [26], so we asked whether mHWTX-IV could act as the same as HWTX-IV. To address this question, we adopted a triple-pulse protocol in which a test pulse (−10 mV) following a strong depolarization (+200 mV, 500 ms) was used to measure available TTX-S sodium currents. 1μM mHWTX-IV most completely inhibited inward TTX-S sodium current induced by first pulse. After a strong depolarization, inward sodium current did not induce by a test −10 mV pulse, it implied that mHWTX-IV still bound to the channel even extremely depolarization (Fig. 7A). This is similar to the action of CcoTx2 on Nav1.2 channel and JZTX-IX on DRG neuron [27], [28].

### Effects of mHWTX-IV on Nav1.7

To confirm whether the current could elicit by high depolarization after application of HWTX-IV or mHWTX-IV, the same triple-pulse protocol was also used to measure available currents. Depolarization to +50, +100 mV for 500 ms induce little recovery of Nav1.7 current which inhibited by both HWTX-IV and mHWTX-IV. When the voltage increased to +150, +200 mV, the current inhibited by HWTX-IV was recovered about 34% and 78%. While no obvious current was elicited at +150 or +200 mV after application of mHWTX-IV (Fig.7B). These data indicate that mHWTX-IV strongly bind to voltage sensor of sodium channel even at extreme depolarization.

## Discussion

Here we have described the purification and characterization of a posttranslational modified peptide toxin, mHWTX-IV, from the Chinese bird spider, *Ornithoctonus huwena* Wang. Mass spectrometry was used to determine the amino sequence of mHWTX-IV and to demonstrate that pyroglutamic acid is at the N-terminus. This peptide contains an N-terminal posttranslational modification, pyroglutamic acid, not previously reported in the spider toxins. It is also the first report demonstrating that the modification increases the toxin's ability to trap the voltage sensor of sodium channel [29], [30].

Our interest in these two peptides was initially stimulated because of their 18 Da difference in molecular mass. Mass spectral sequence studies of the two toxins showed that the difference occurred in the first two N-terminal residues, Glu or Cys. Since the mass of mHWTX-IV increased by 342 Da after reduction and alkylation of cysteine, the cysteine is behaving like a normal thiol, and consequently it the modification must be at the N-terminal glutamic acid. Coincidentally at about the same time, Macintosh reported on a conotoxin that was unreactive to Edman degradation and concluded that it was N-terminally blocked with pyroglutamic acid [22]. Likewise, the cyclization of Glu with loss of 18 Da, and the lack of reactivity on Edman degradation indicates that the N-terminus of the novel toxin, mHWTX-IV, is pyroglutamic acid.

It appears that modification of toxins is widespread, having been found especially in conotoxins, and *Conus* venom peptides are proving to be among the most highly post-translationally modified gene products known [31]. The finding of a glutaminyl cyclization in conotoxin bromoheptapeptide was the first report of such a reaction in *Conus* venom, but no further study about the function has been reported [22]. In contrast to the toxins, N-terminal pGlu is quite common in peptides and proteins in animals are common although the role (if any) of the pGlu residue in these cases is unclear. Depending on the peptide, the role of pGlu could be either to stabilize against degradation by aminopeptidases, e.g., as in the cases of GnRH and MCP-2 [32], [33], or to influence interactions between the peptide and specific receptors, e.g., TRH [34]–[36].

Binding of HWTX-IV, α-scorpion and ProTX-II to sodium channel can be reversed by strong depolarization, indicating that voltage sensor activation can reverse toxin binding [11], [19], [20]. mHWTX-IV is the first reported spider peptide having pyroglutamic acid at the N-terminus. We also demonstrated that the modification did not change the function of HWTX-IV but enhanced its binding strength to the sodium channel. HWTX-IV binds with the S3–S4 linker D816 and E818 in domain II of Nav1.7 by electrostatic interaction, and the N-terminal modification reported here could strengthen the interaction by removing a negative residue [11]. This proposed mechanism is consistent with another known function, namely that pyroglutamic acid stabilizes proteins by compensating for the loss of N-terminal basicity caused by glutaminyl cyclization [29].

N-terminal generation of pyroglutamic acid is a major posttranslational modification of secretory proteins and peptides in animals. Frequently, the moiety is important in exerting biological function in either mediating interaction with receptors or stabilizing against N-terminal degradation [29]. Our work has presented for the first time evidence demonstrating that N-terminal generation of pyroglutamic acid of a peptide neurotoxin can enhance the binding ability to its ion-channel target. Thus, this peptide could be an important tool for studying posttranslational modification and the effect of the modification on function.

## Supporting Information

Figure S1 N-terminal sequences of HWTX-IV were determined by Endman degradation. Residue signals were detected in each panel and six residues were identified.
(TIF)

Figure S2 Sequences of mHWTX-IV were determined by Endman degradation. No obvious signal of amino acid residue was observed in the panel (residue 1-residue 6).
(TIF)
