# Supplementary figures and images for: Native Pyroglutamation of Huwentoxin-IV: A Post-Translational Modification that Increases the Trapping Ability to the Sodium Channel
Source: PLoS One. 2013 Jun 24;8(6):e65984. doi: 10.1371/journal.pone.0065984 (PMC3691182; doi:10.1371/journal.pone.0065984)

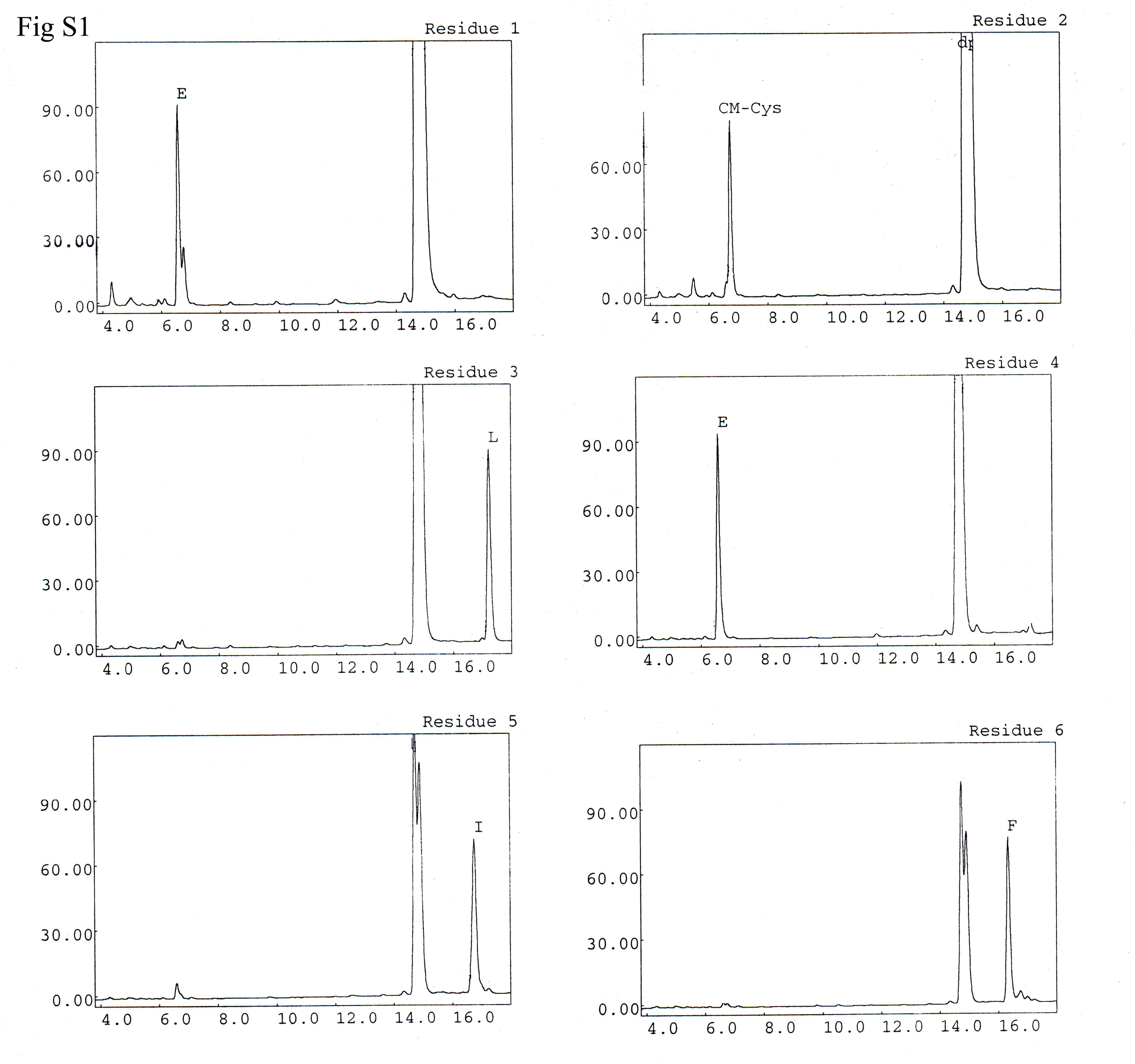

Supplement: Figure S1 — N-terminal sequences of HWTX-IV were determined by Endman degradation. Residue signals were detected in each panel and six residues were identified. (TIF) [file pone.0065984.s001.tif]

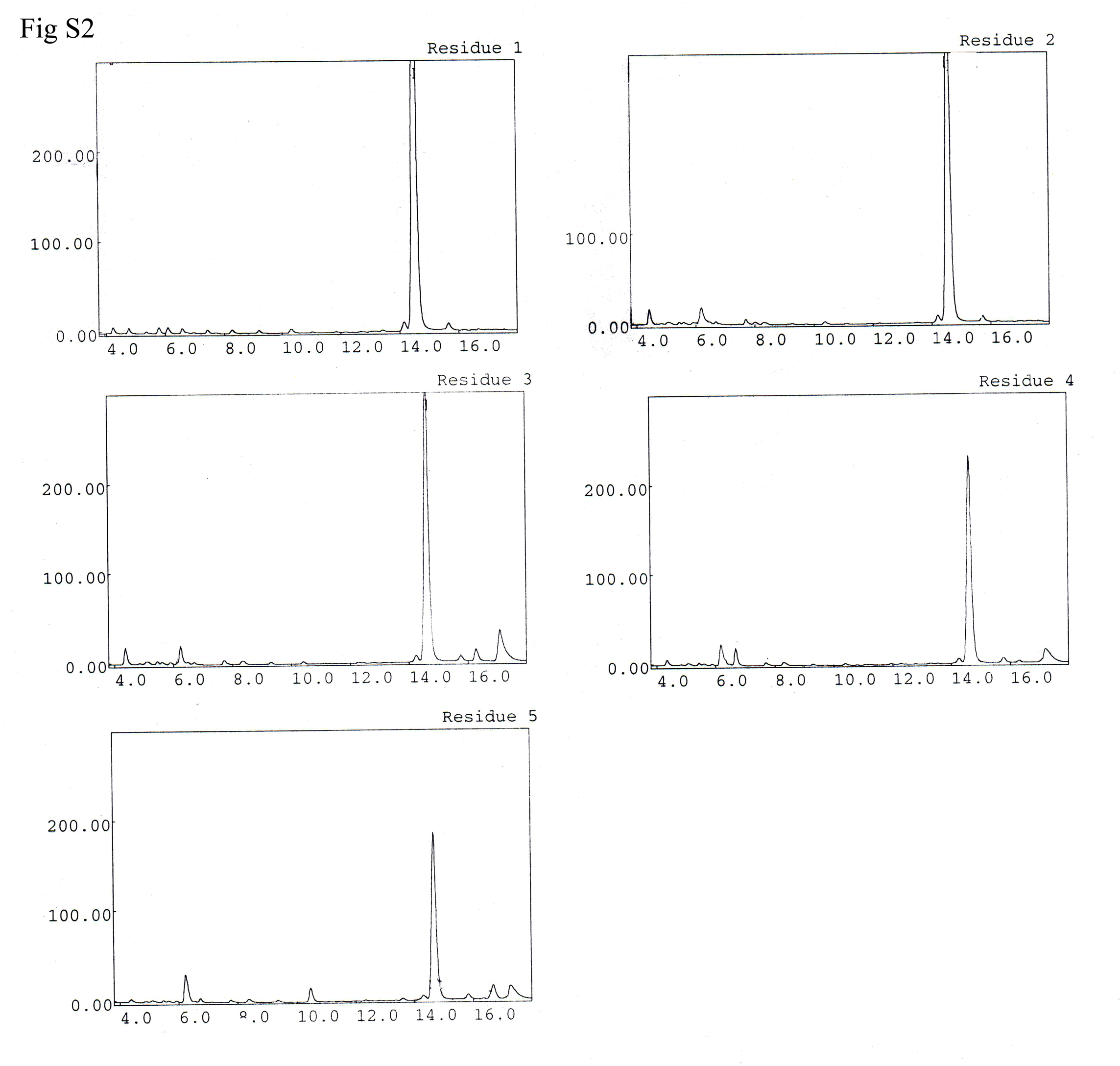

Supplement: Figure S2 — Sequences of mHWTX-IV were determined by Endman degradation. No obvious signal of amino acid residue was observed in the panel (residue 1-residue 6). (TIF) [file pone.0065984.s002.tif]
